# Monospecific Biofilms of *Pseudoalteromonas* Promote Larval Settlement and Metamorphosis of *Mytilus coruscus*

**DOI:** 10.1038/s41598-020-59506-1

**Published:** 2020-02-13

**Authors:** Li-Hua Peng, Xiao Liang, Jia-Kang Xu, Sergey Dobretsov, Jin-Long Yang

**Affiliations:** 1 International Research Center for Marine Biosciences, Ministry of Science and Technology, Shanghai Ocean University, Shanghai, China; 2 Key Laboratory of Exploration and Utilization of Aquatic Genetic Resources, Ministry of Education, Shanghai Ocean University, Shanghai, China; 3 National Demonstration Center for Experimental Fisheries Science Education, Shanghai Ocean University, Shanghai, China; 4 Department of Marine Science and Fisheries, College of Agricultural and Marine Sciences, Sultan Qaboos University, Muscat, Oman; 5 Center of Excellence in Marine Biotechnology, Sultan Qaboos University, Muscat, Oman

**Keywords:** Ecology, Ecology

## Abstract

As a stage of life cycle, larval settlement and metamorphosis are critical processes for persistence of many marine invertebrate populations. Bacterial biofilms (BFs) could induce larval settlement and metamorphosis. *Pseudoalteromonas*, a widely distributed genus of marine bacteria, showed inductive effects on several invertebrates. However, how *Pseudoalteromonas* BFs induce settlement and metamorphosis of *Mytilus coruscus* remains unclear. *Pseudoalteromonas marina* BFs with the highest inducing activity were further investigated to define inductive cues. Surface-bound products of *P. marina* BFs could induce larval settlement and metamorphosis. *P. marina* BFs treated with formalin, antibiotics, ultraviolet irradiation, heat and ethanol significantly reduced inductive effects and cell survival rates. The confocal laser scanning microscopy and the biovolume analysis showed the dominance of α-polysaccharides on *P. marina* BFs. Treatment of BFs with amylases, proteases and lipase led to the decrease of inducing activity, suggesting that inductive cues of *P. marina* BFs may comprise of molecular domains of polysaccharides, proteins, and lipids. Finding inductive cues of BFs could put forward further studies about the mechanism of larval settlement and metamorphosis of marine invertebrates.

## Introduction

Most marine benthic invertebrates have a planktonic larval phase in their life cycle, which plays an important role in their survival and development^1,2^. Competent larvae choose an acceptable substratum to settle and metamorphose to the benthic stage^3,4^. This critical step is affected by abiotic factors (exogenous physical and chemical) and biotic factors (endogenous and exogenous)^2,4–6^. Natural biofilms (BFs) which developed in the sea have a complex structure and are composed of many species of microorganisms^7^.

Natural BFs were proposed as one of biotic cues to stimulate larval settlement and metamorphosis^8–10^. In benthic communities, bacteria exist in a form of a bacterial BF, which is a prevalent microbial lifestyle^11,12^. Not only multispecies BFs but also monospecies BFs of bacteria can induce larval settlement and metamorphosis^13,14^. It has been shown that BFs of *Pseudoalteromonas*, *Vibrio*, *Shewanella* and *Tenacibaculum* can induce larval settlement and metamorphosis^3,14–16^.

*Pseudoalteromonas* species are widely distributed at various marine environments and have high ecological significance^17,18^. BFs of some *Pseudoalteromonas* species were reported as inducers^14,19,20^ or inhibitors^21–24^ of larval settlement. However, it is unclear whether varying species of *Pseudoalteromonas* show inducing or inhibiting activity to the mussel *Mytilus coruscus*.

Bacteria in BFs were buried in extracellular polymeric substances (EPS) which are mostly secreted by bacterial strain itself^25^. Biofilm’s EPS include fatty acids, proteins, polysaccharides, nucleic acids and other biopolymers^25^. EPS could be divided into the water-soluble part and water-insoluble part^25–28^. The water-soluble and -insoluble cues were involved in larval settlement and metamorphosis of marine invertebrates, respectively^29^. In *M. coruscus*, both water-borne and surface-bound cues are need for *Shewanella* sp. 1 BFs to induce larval settlement and metamorphosis^14^. However, how EPS and its components affected inductiveness of *Pseudoalteromonas* BFs remain unclear.

*M. coruscus* is distributed at the temperate zone of coastal waters in East Asia. It is a common economic shellfish and a major fouling organism in China^30^. Thus, investigation of *M. coruscus* settlement has a great ecological and industrial significance^30^.

In the present study, inducing activities of seven *Pseudoalteromonas* strains were tested. To elucidate inductive cues, *P. marina*, which had the highest inducing activity was used as a model strain. In this study we investigated: (1) larval responses to varying species of *Pseudoalteromonas* in order to examine whether *Pseudoalteromonas* BFs showed the similar inducing or inhibiting activity to the mussel *M. coruscus*; (2) whether the surface-bound products of BFs have inducing activity; (3) the inducing activity and cell mortality of BFs treated with formalin, ethanol, heat and ultraviolet irradiation, in order to determine properties of BF inductive cues; and (4) the inductive effect of BFs treated by amylases, proteases, and a lipase to determine bioactive moieties of inductive cues.

## Results

### Inducing activity of *Pseudoalteromonas* biofilms

**Figure 1:**
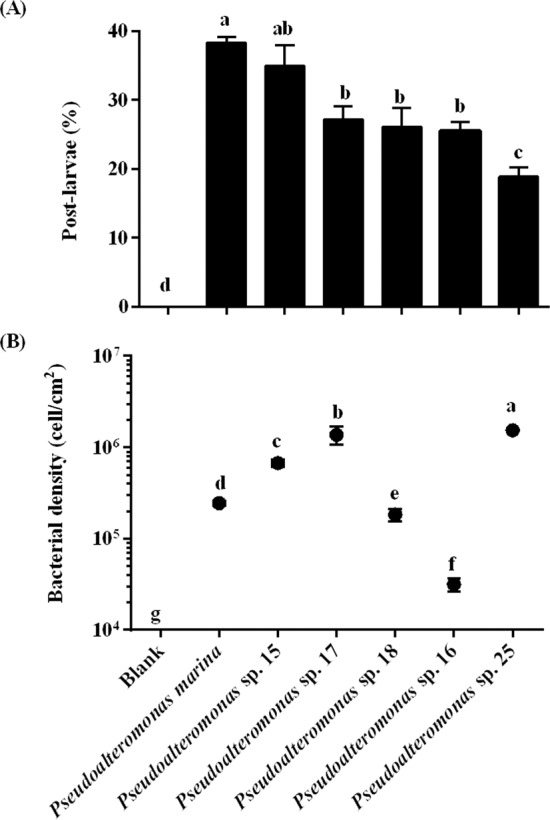
*Pseudoalteromonas* biofilms induced larval settlement and metamorphosis of *M. coruscus*. (**A**) Inducing activity of *Pseudoalteromonas* BFs on larval settlement and metamorphosis of *M. coruscus*. (**B**) The bacterial densities of *Pseudoalteromonas* BFs (n = 30). Values that are significantly different between each other at *p* < 0.05 are indicated by different letters above the bars or the dots. Data are means ± SE (n = 9).

**Figure 2:**
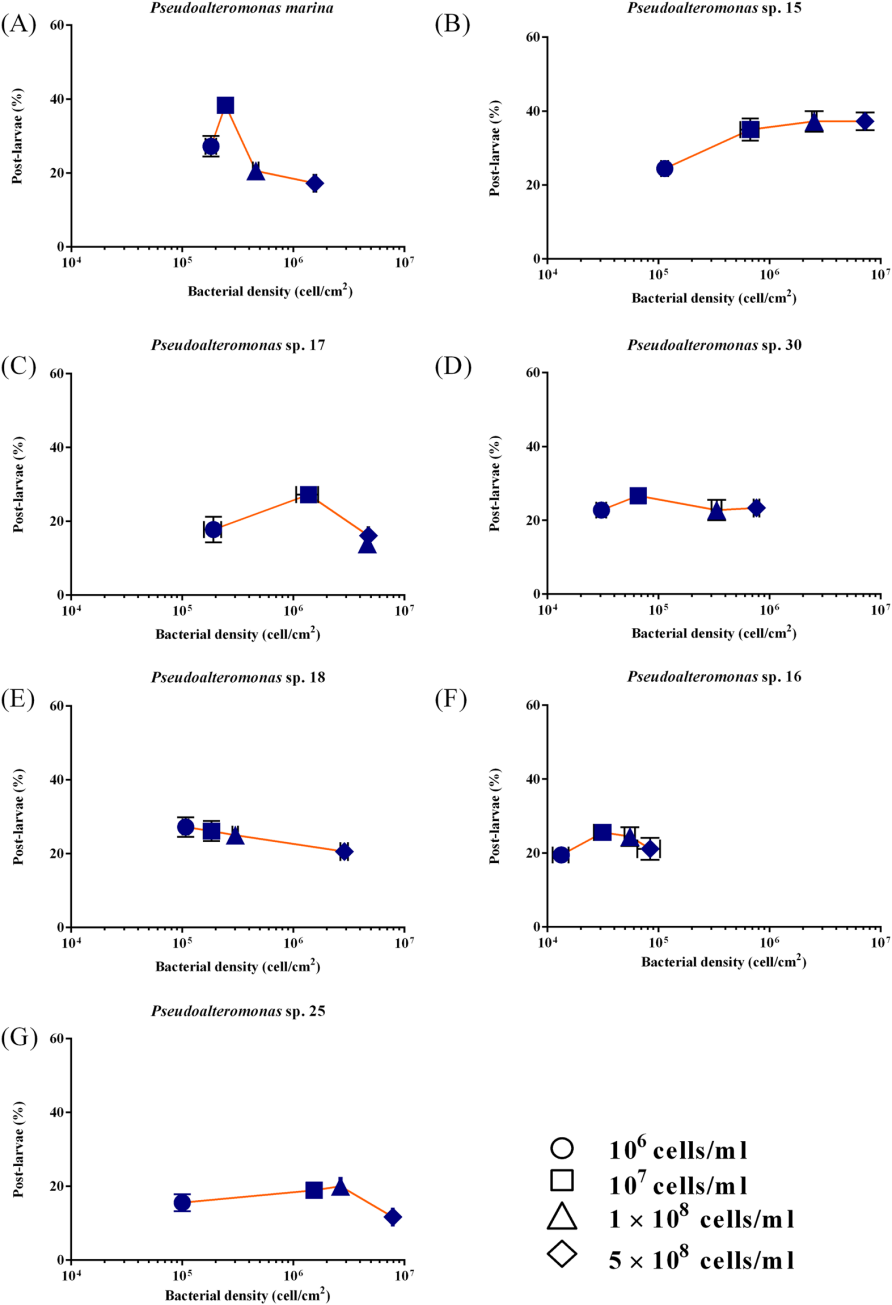
Percentages of *M. coruscus* post-larvae on monospecific bacterial biofilms of various densities after 48 h. (**A**) *Pseudoalteromonas marina*; (**B**) *Pseudoalteromonas* sp. 15; (**C**) *Pseudoalteromonas* sp. 17; (**D**) *Pseudoalteromonas* sp. 30; (**E**) *Pseudoalteromonas* sp. 18; (**F**) *Pseudoalteromonas* sp. 16; (**G**) *Pseudoalteromonas* sp. 25. Data are the mean percent (±SE) of nine replicates. Bacterial densities are the means (±SE) of 30 random fields of view counts.

Seven difference species of the genus *Pseudoalteromonas* were isolated from nature BFs and mussel’s gut (Table S1) and their phenotypes are shown (Fig. S1). All bacteria showed inducing activity (20%–40%) in comparison to the negative control (*χ*^2^: d*f = *6, *p* < 0.001, Fig. 1A). Among tested bacteria, *Pseudoalteromonas marina* BFs showed the highest inducing activity (38.33 ± 0.83%), while *Pseudoalteromonas* sp. 25 BFs showed the lowest inducing activity (11.67 ± 0.83%) (Fig. 1A). *Pseudoalteromonas* sp. 25 BFs had higher cell density (1.50 ± 0.05 × 10^6^ cells cm^−2^) than that of *P. marina* BF (2.40 ± 0.18 × 10^5^ cells cm^−2^) (Fig. 1B). The inducing activity of *Pseudoalteromonas* sp. 17 BFs, *Pseudoalteromonas* sp. 18 BFs, *Pseudoalteromonas* sp. 30 BFs, and *Pseudoalteromonas* sp. 16 BFs showed no significant (*p* > 0.05) difference (Fig. 1A), while the densities of these four strains were significantly different (Fig. 1B). The effect of bacterial density of *P. marina* (Fig. 2A), *Pseudoalteromonas* sp. 15 (Fig. 2B), *Pseudoalteromonas* sp. 17 (Fig. 2C), *Pseudoalteromonas* sp. 30 (Fig. 2D), *Pseudoalteromonas* sp. 18 (Fig. 2E), *Pseudoalteromonas* sp. 16 (Fig. 2F), *Pseudoalteromonas* sp. 25 (Fig. 2G) on larval settlement and metamorphosis was different. The significant correlations were observed between bacterial cell densities and the inducing activity of *P. marina*, *Pseudoalteromonas* sp. 15, and *Pseudoalteromonas* sp. 17 BFs (Table 1).

**Table 1:** Correlation analyses between bacterial density of monospecific bacterial biofilms and their inducing activity. r = Spearman’s rank order correlation analysis; *p*: *p*-value; *: significant values at *p* < 0.05.

Tested bacteria	Bacterial density	
	r	*p*
*Pseudoalteromonas marina*	−0.5334	0.0008^*^
*Pseudoalteromonas* sp. 15	0.5404	0.0007^*^
*Pseudoalteromonas* sp. 17	−0.568	0.0003^*^
*Pseudoalteromonas* sp. 30	−0.0935	0.5875
*Pseudoalteromonas* sp. 18	−0.3197	0.0573
*Pseudoalteromonas* sp. 16	0.0147	0.932
*Pseudoalteromonas* sp. 25	−0.2048	0.2308

### Inductive effects of surface-bound products

**Figure 3:**
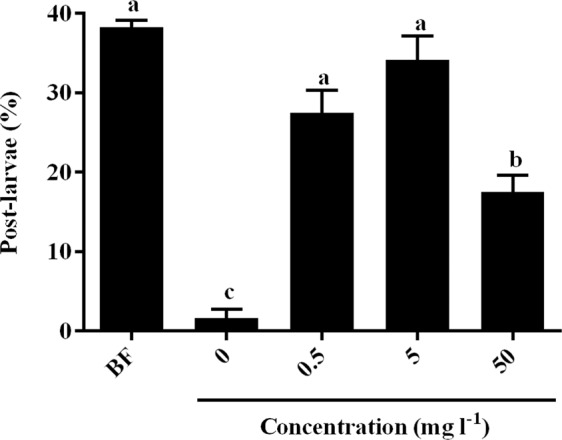
Percentages of post-larvae after exposure to surface-bound products of *P. marina* BF for 48 h. BF = *P. marina* BF. Letters indicated significant differences (*p* < 0.05). Data for post-larvae (%) are the means (+SE) of six replicates.

**Figure 4:**
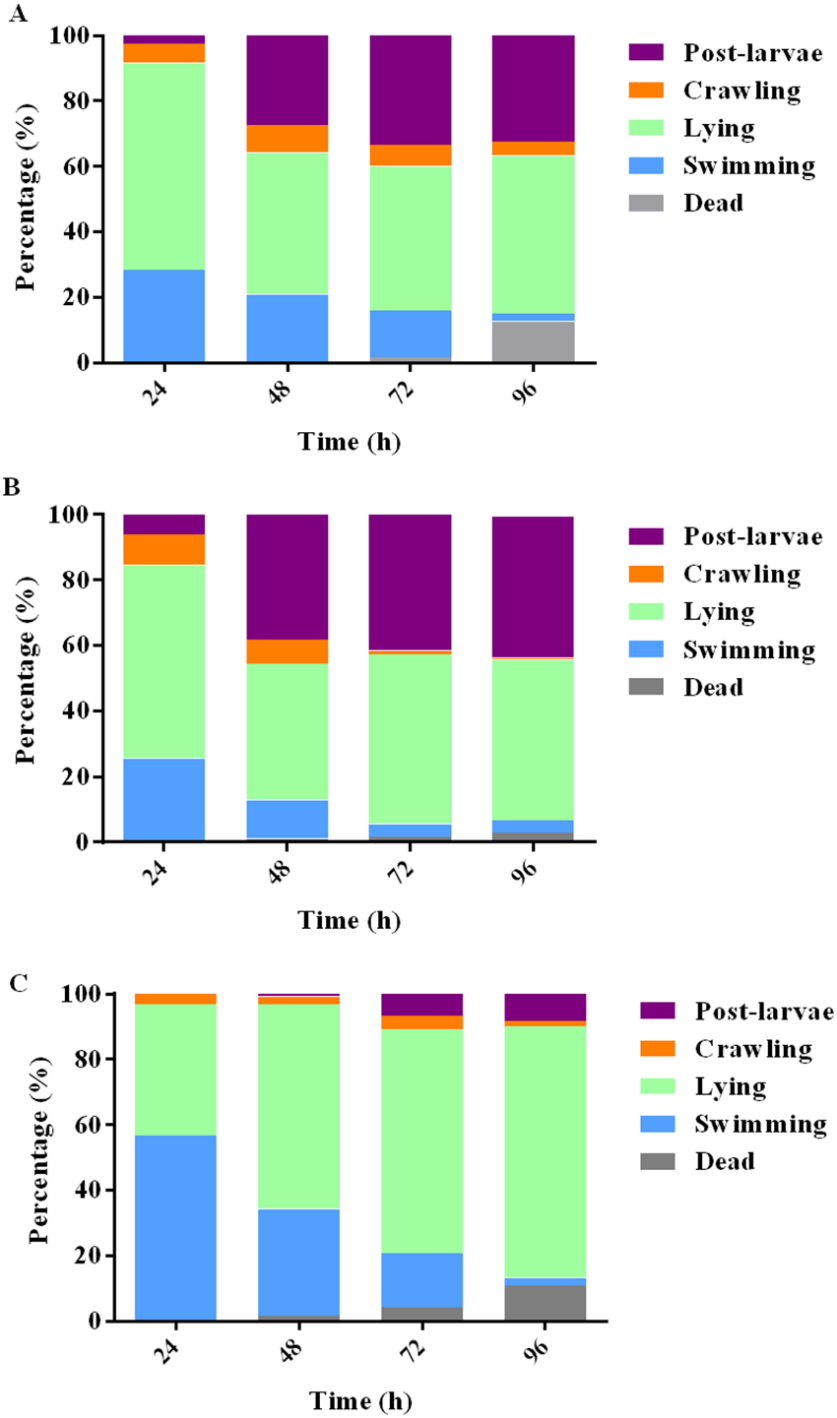
Behavior of larvae (swimming, crawling, lying, dead, post-larvae) when exposed to: (**A**) 0.5 mg l^−1^ of surface-bound products of *P. marina* BFs, (**B**) BFs of *P. marina* and (**C**) autoclaved filtered seawater. The data are expressed as percentages.

The surface-bound products of *P. marina* BFs significantly promoted larval settlement and metamorphosis at all tested concentrations (*χ*^2^: d*f = *4, *p* < 0.001, Fig. 3). The inducing activities of surface-bound products at concentrations of 0.5 mg l^−1^ and 5 mg l^−1^ were similar to that of *P. marina* BF. The surface-bound products at the concentration 50 mg l^−1^ were less inductive than *P. marina* BF (Fig. 3). After 24 h, 28% and 25% larvae were swimming in the presence of 0.5 mg l^−1^ of surface-bound products (Fig. 4A) and BFs (Fig. 4B), while more than 50% of larvae were swimming in autoclaved filtered seawater (AFSW, Fig. 4C). After 48 h, 28% post-larvae were observed in the presence of the 0.5 mg l^−1^ of surface-bound products (Fig. 4A). In the presence of *P. marina* BFs, the percentages of swimming and crawling larvae decreased over time and the post-larvae reached 38% after 48 h (Fig. 4B).

### Inducing activity of treated *P. marina* biofilms

**Figure 5:**
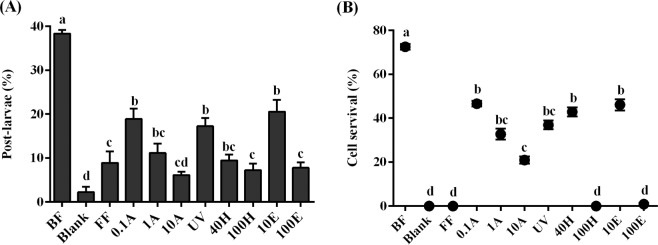
The inducing activity and cell survival of *P. marina* biofilms (BFs). (**A**) Percentages of post-larvae of *M. coruscus* on treated BFs; (**B**) Cell survival (%) of treated BFs. BF = *P. marina* biofilm; Blank = clean glass slide; FF = bacterial BFs treated with 5% formalin; 0.1 A, 1 A, and 10 A = bacterial BFs treated by antibiotic solutions. (0.1 A = 0.1 mg ml^−1^ each of penicillin G potassium and streptomycin sulfate, 1 A = 0.1 A concentration increased tenfold, 10 A = 0.1 A concentration increased hundredfold); UV = bacterial BFs treated with UV irradiation (5 W m^2^, 1 h); 40 H and 100 H = bacterial BFs heated at 40 °C and 100 °C, respectively; 10E and 100E = bacterial BFs treated with 10% and 100% ethanol, respectively. Letters indicate significant differences (*p* < 0.05). Data are the means (+SE) of nine replicates.

The inducing activity of *P. marina* BFs decreased significantly (*χ*^2^: d*f = *10, *p* < 0.001) after treatment with formalin, antibiotic, ultraviolet irradiation, heat and ethanol (Fig. 5A). Similarly, cells survival significantly (*χ*^2^: d*f = *10, *p* < 0.001, Fig. 5B) decreased. The FF treatment led to low inducing activity and 100% cell mortality in *P. marina* BFs (Fig. 5). The antibiotic treatment significantly changed cell survival and inductiveness of BFs (*p* < 0.05) (Fig. 5). No cell survived after the treatment with the highest treatments of 100E and 100 H (Fig. 5B).

### CLSM images and enzyme treatment of *P. marina* biofilms

**Figure 6:**
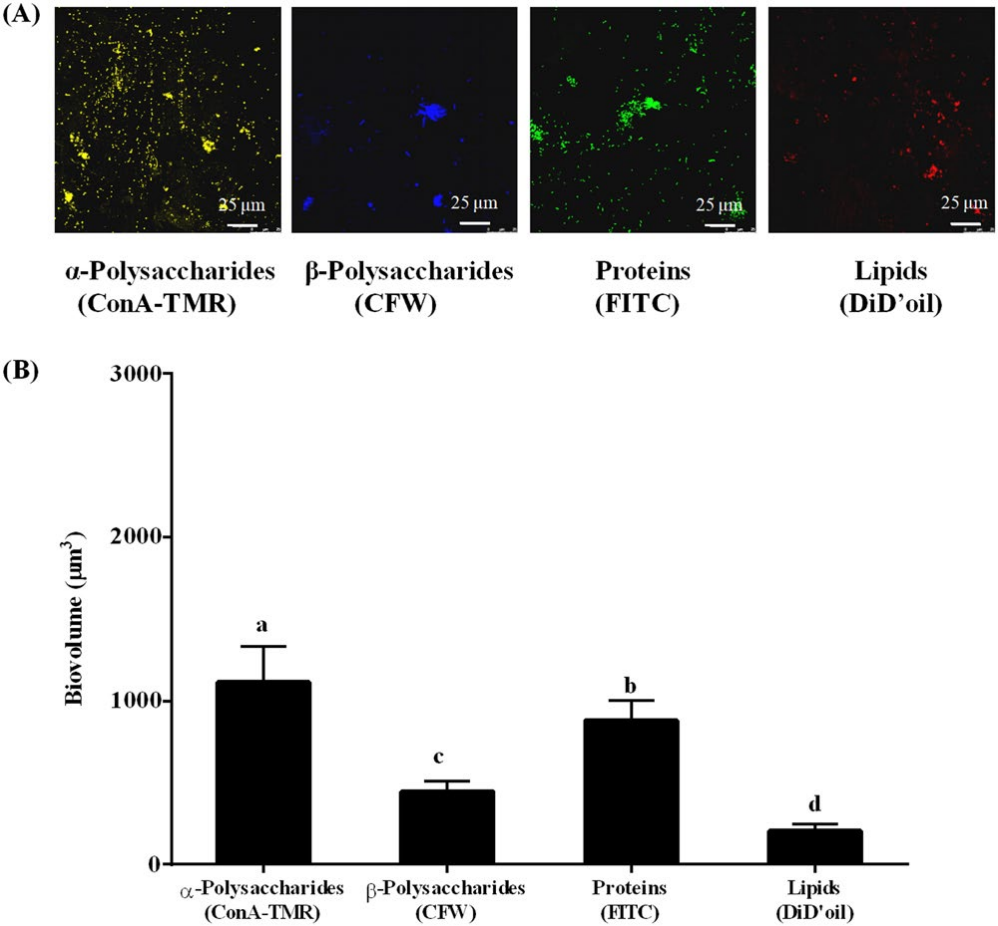
The analysis of CLSM images of *P. marina* biofilms. (**A**) The distribution and (**B**) biovolume of α-polysaccharides, β-polysaccharides, proteins and lipids on BF. ConA-TMR is concanavalin A, tetramethylrhodamine conjugate; FITC is fluorescein isothiocyanate isomer; CFW is calcofluor white M2R; DiD’oil is DiIC 18 (5) oil, 1,1′-dioctadecyl-3,3,3′,3′-tetramethylindodicarbocyanine perchlorate. Data is the mean (+SE) of nine replicates.

**Figure 7:**
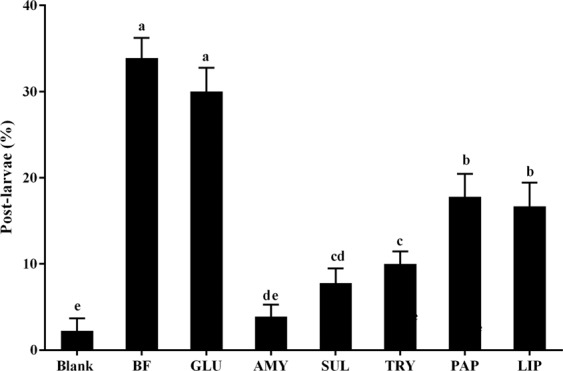
Percentages of post-larvae of *M. coruscus* on treated *P. marina* biofilms. Blank = a clean glass without BF; BF = *P. marina* biofilms; AMY = biofilms treated with α-amylase, SUL = biofilms treated with sulfatase, TRY = biofilms treated with trypsin, PAP = biofilms treated with papain, LIP = biofilms treated with lipase. Data are the means (+SE) of nine replicates.

As shown by scanning electron microscope (SEM) images, the bacterial morphology and distribution of *P. marina* BFs was shown in Fig. S2. The CLSM images showed extracellular α-Polysaccharides, β-Polysaccharides, proteins and lipids of *P. marina* biofilms (Fig. 6A). The biovolumes of α-polysaccharides reached up to 1178 ± 222 μm^3^ and was higher than that of β-polysaccharides, proteins and lipids (Fig. 6B). The percentages of post-larvae decreased significantly (*p* < 0.05) on *P. marina* BFs treated with α-amylase (AMY), sulfatase (SUL), trypsin (TRY), papain (PAP) and lipase (LIP) (Fig. 7). The percentages of post-larvae showed no significant change (*p* > 0.05) when BFs were treated with β-glucuronidase (GLU) (Fig. 7).

## Discussion

*Pseudoalteromonas* gained attention due to its ecological significance^18,23^. Bacteria belonging to *Pseudoalteromonas* genus have been commonly observed in marine BFs^17^. *Pseudoalteromonas* species induce larval settlement of the polychaete *Hydroides elegans*^19^ and the mussel *M. coruscus*^14^ and prevent settlement of bacteria and fungi, algal spores and invertebrate larvae^23,24^. In present study, BFs of seven different *Pseudoalteromonas* species could induce larval settlement and metamorphosis of *M. coruscus*.

The inducing activity of *Pseudoalteromonas* species isolated from natural BFs and the gut of the mussel showed similar inducing activities. This indicates that there is no relation between bacterial origin and inducing activity of settlement. Similar results were observed for other invertebrate species^19,21^.

Previously, it has been shown that bacterial densities of BFs were positively^31–33^ or negatively^34,35^ correlated with larval settlement of *H. elegans*, *Mytilus edulis*, *M. galloprovincialis*, *Pocillopora damicornis*, *Bugula neritina* and *Amphibalanus (*= *Balanus) amphitrite*. Here, the correlation between bacterial cell density of *Pseudoalteromonas* BFs and their inductive activity varied among the species. This fact could suggest that interactions between bacterial BFs and larvae of *M. coruscus* are different from other *Mytilus* species. Our results are consistent with the previous report on *M. coruscus*^14^.

Our study showed that surface-bound products of *P. marina* BFs could trigger the similar percentages of crawling behavior of *M. coruscus* as BFs of this bacterium. In contrast, most of larvae keep swimming in AFSW. Larval response to inductive cues with crawling behavior was also shown for other species such as *M. galloprovincialis*^36,37^. Similarly, the polychaete larvae of *H. elegans* stop swimming and start crawling within a few minutes of exposure to inductive bacterial BFs^5^. In addition, the crude extracts of surface-bound products of *Alteromonas* sp. 1 BFs could not induce larval metamorphosis of *M. galloprovincialis*^37^. Surface-bound products of *P. marina* BFs could induce larval metamorphosis of *M. coruscus*. This difference between *M. galloprovincialis* and *M. coruscus* might be due to the different species or composition of surface-bound products. *Alteromonas* sp. 1 BFs scraped off from Petri dishes were sonicated for 5 min and the crude extracts of surface-bound products contained both surface-bound products and bacterial cellular contents^37^. In present study, *P. marina* BFs scraped off from Petri dishes were mixed with PBS by pipetting instead of sonication and crude extracts of surface-bound products had less cellular contents. On the other hand, surface-bound products in the present study contained only compounds >3.5KDa, while inductive cues in other studies had different molecular weights, such us, 1–10 KDa (scyphozoan)^15^, ≤0.3 KDa (oyster)^38^ and ≤3 KDa (*M. galloprovincialis*)^37^.

It is shown that water-borne cues could induce larval metamorphosis^29^ of *Phestilla*^39^, *Adalaria proxima*^40^, and *Balanus amphitrite*^41^ as well as larval settlement of oysters^42,43^. For *Mytilus* species, like *M. coruscus*^14^ and *M. galloprovincialis*^37^, water-borne cues could induce larval settlement but not metamorphosis. Similarly, physical properties of natural BFs, instead of water-borne cues, induced settlement of a pearl oyster^44^. Thus, in the present study, we investigated the effect of surface-bound cues.

Previous studies have shown that biofilms of bacteria isolated from surface of adult *B. amphitrite*^41^ and *M. coruscus*^14^ could induce larvae metamorphosis. In the present study, bacteria carried by larvae showed no inductive effect on larval settlement and metamorphosis, it may be due to that low numbers of bacteria could not form an effective biofilm. Bacterial matrix could promote biofilm formation^45^. Thus, in the presence of surface-bound products, biofilms being seeded with bacteria from larvae might form easier and show inducing activity. Thus, the inducing activities of biofilms seeded by larvae need to be examined in the future.

To investigate characteristics of inductive cues, *P. marina* BFs were treated by antibiotics, formalin, ethanol, heat, and UV. All treatments led to the decrease of the inductive activity of the *P. marina* BFs and the increase of the mortality of bacteria. Our results were consistent with previous research with larvae of *M. galloprovincialis*^33,37^ and *M. coruscus*^14^. Our and previous studies^14,33,37^ suggested that inductive cues were susceptible for above-mentioned treatments, and the viability of bacterial cells was important for the production of inductive cues. Similar results were obtained in experiments with treated BFs and other larvae of marine invertebrates^46–48^. For example, formalin-killed BF of *Pseudomonas marina* induced low settlement and metamorphosis (less than 9%) of *Janua*^46^. Natural BFs treated with ethanol (5% and 10%), heat (40 °C) led to the reduction of inducing activity of larval metamorphosis of sea urchins^47^. Antibiotic treatment reduced the percentages of larvae settled on *Roseobacter* and *Proteobacteria* BFs^48^.

Analysis of CLSM images showed that *P. marina* BFs had the higher biovolume of the α-polysaccharides than those of proteins, β-polysaccharides, and lipids. In opposite, the biovolume of α-polysaccharides was lower than those of β-polysaccharides and proteins in BFs of *Salmonella enterica* serotype Agona^49^. This suggested that components of BFs varied between bacterial species.

In order to investigate the inductive cue from *P. marina* further, BFs were treated with different enzymes. Treatment of BFs with α-amylase reduced its inductiveness. This suggested that exopolysaccharides or glycoproteins are involved in larval metamorphosis of *M. coruscus*. Exopolysaccharides or glycoproteins of BFs bound to lectins were involved in larval settlement and metamorphosis of *M. galloprovincialis*^37^ and *Janua (Dexiospira) brasiliensis*^50^. Glycoproteins were involved in larval settlement of marine invertebrates. For example, the settlement-inducing protein complex, a glycoprotein consisting of lentil lectin-binding sugar chains, induced larval settlement of *B. amphitrite*^51,52^. A tri-peptide with arginine at the C-terminal was inductive for *Crassostrea virginica*^53^, and glycoproteins in the organic matrix of pacific oyster shells were inductive for *Crassostrea gigas* settlement^54^. A chemical identity of bacterial exopolysaccharide or glycoprotein that induced *M. coruscus* larval settlement requires further investigations.

Moreover, proteases (trypsin and papain) reduced inductive activity of *P. marina* BFs, suggesting that the bioactive moieties of inductive cues were proteinaceous. Earlier studies have proved that proteins could induce larval settlement or metamorphosis of the oyster^54^, jellyfish^55^ and barnacles^56^. However, purified proteins inducing larval settlement and metamorphosis of *M. coruscus* are still unknown.

The result of lipase treatment suggests that lipids may be involved in larval settlement and metamorphosis. In previous studies, fatty acids were suggested as chemical cues for larval settlement or metamorphosis. For example, free fatty acids isolated from a coralline red alga induced settlement and metamorphosis of *Pseudocentrotus depressus* and *Anthocidaris crassispina*^57^. Free fatty acids isolated from sand matrix of adult tubes of polychaetes induced larval settlement and metamorphosis of *Phragmatopoma* species^58,59^. For barnacle larvae, lipids played important roles in permanent adhesive by creating a supportive environment and reducing bacterial biodegradation of nascent adhesive plaque^60^. The role of lipids in settlement and metamorphosis of *M. coruscus* still needs to be researched.

In summary, all tested *Pseudoalteromonas* species could promote *M. coruscus* larval settlement and metamorphosis. Surface-bound products of biofilms contained inductive cues. The inductive cues were susceptible to formalin, ethanol, heat and ultraviolet irradiation treatments. The viability of bacterial cells was important for the production of inductive cues. The inductive cues induced larval settlement and metamorphosis may include polysaccharides, proteins and lipids.

## Materials and Methods

### Bacterial isolation and cultivation

Bacteria were isolated from four weeks old natural BFs and gut of the wild two-year adult mussel *M. coruscus*. Suspensions of natural BFs were prepared as described in the previous study^19^. Briefly, natural BFs which formed on clean glass at seawater of China East Sea (122°46′ E; 30°43′ N), were scraped off from the glass slides and suspended into autoclaved filtered seawater (AFSW). The adult mussels were collected from the same area, washed three times by AFSW and dissected on ice by sterilized tools. The gut tissues were pounded and suspended in AFSW. The suspensions of natural BFs and gut cells were carried out 10-fold serial dilutions to the dilution of 10^4^ times with AFSW. Individual bacteria were separated by spreading diluted cell suspension on Zobell 2216E agar plates. One liter of Zobell 2216E agar plates contained 5.0 g peptone, 1.0 g yeast extract, 0.01 g Ferric citrate and 15.0 g agar. All reagents were purchased from Sigma Company (United States). Individual bacterial colonies were selected based on their shape, morphology and colour. Individual colonies were purified three times through plate streaking. Prior to the experiments, all bacterial strains were stored at −80 °C with cryoprotectant (0.9% physiological saline, 15% glycerol).

### Identification of *Pseudoalteromonas*

Prior to identification, bacterial strains were cultured in Zobell 2216E medium for 16–24 h. After centrifugation, broth culture was discarded and cells were collected for DNA extraction. TIANamp Bacteria DNA Kit was used for genomic DNA extraction following manufactures protocol (TIANGEN Biotech Company, Beijing, China). The primers 27 F ((5′-AGA GTT TGA TCC TGGCTC AG-3′) and 1492 R (5′-GGT TAC CTT GTT ACGACT T-3′) were used to specifically amplify the 16 S rRNA gene sequence following a modified PCR procedure^61^. The reaction mixture of the amplification contained 0.5 μl of 27 F (10 μM), 0.5 μl of 1492 R (10 μM), 0.5 μl of DNA (100 ng μl^−1^), 0.3 μl of Taq (5 U μl^−1^), 2 μl of MgCl2 (25 mM), 0.5 μl of deoxynucleotide triphosphates (10 mM each), and 20.5 μl of sterilized distilled water. The amplification was performed using the following PCR conditions: an initial denaturing step (94 °C, 5 min), 35 cycles of denaturation (94 °C, 30 s), an annealing (55 °C, 30 s), and an elongation (72 °C, 2 min). An Eppendorf Mastercycler (Eppendorf, Germany) was used. The PCR products were sent to Sangon Biotech Company (Shanghai, China) to get 16 S rRNA gene sequences. These sequences were blasted using the National Center for Biotechnology Information (NCBI) website to identify close matches.

### Preparation of biofilms

The monospecific BFs of different *Pseudoalteromonas* strains were prepared following modified published method^19^. Briefly, *Pseudoalteromonas* strain was cultured in 80 ml of Zobell 2216E medium at 25 °C for 24 h with shaking (200 rpm). The bacterial cells were harvested from culture by centrifugation (1600 g, 15 min). Cells were re-suspended in AFSW and washed thrice to remove broth residues. Finally, cell pellets were re-suspended in AFSW again as a bacterial stock solution. One ml diluted bacterial stock solution (1:1000) was stained for 5 min with acridine orange solution (AO, 0.1%) and then transferred to a 0.22 μm Nuclepore Track-Etch Membrane (Whatman, UK) by negative-pressure filtration (diameter of filter mesh: 1.0 cm). The number of bacteria on a membrane was counted in 20 randomly selected fields of view under 1000 × magnification of Olympus BX51 (wavelengths of excitation/emission (Ex/Em) = 490/519 nm)^62^. Bacterial density was re-calculated to cells ml^−1^ using Eq. (1).

1 $$S=C\times \pi \times {(D/2)}^{2}\div{\rm{V}}\times {10}^{3}$$

where *S* was concentration of stock solution (cells ml^−1^); *C* was cells of field of view (cells cm^−2^); *D* was diameter of filter mesh; V was 1 ml which the volume of diluted bacterial stock solution transferred to membrane.

The stock solution of each tested *Pseudoalteromonas* strain was diluted with AFSW to prepare four initial concentrations of bacterial suspension (1 × 10^6^, 1 × 10^7^, 1 × 10^8^ and 5 × 10^8^ cells ml^−1^). Twenty milliliters of bacterial suspension and a microscopic glass slide (2.5 cm × 3.8 cm) was added to a sterilized glass Petri dish (ASONE Corporation, Japan). The Petri dishes were incubated at 18 °C for 48 h until BF was developed. Unattached bacteria were removed by washing the dishes with AFSW three times. The monospecific bacterial BF was the film remained on a glass slide.

### Detection of bacterial density of biofilms

BFs were soaked at 5% formalin solution for 48 h. Fixed BFs were washed with AFSW thrice to remove residual formalin and stained by AO (0.1%, v/v) for 5 min. As mentioned above, bacterial density (cells cm^−2^) was counted using an Olympus BX51 microscope at 1000 × magnification and 10 fields of views. The assay had three independent replications.

### Fertilization and larval culture

Two years old of *M. coruscus* were collected from Shengsi, Zhoushan, China (122°44′ E; 30°73′ N). Fertilization was performed based on the methods of Yang *et al*.^63,64^. Clean mussels were incubated on ice overnight. Then, mussels were put into 10 liters polycarbonate tanks. Spawning was stimulated by addition of *ca* 21 °C filtrated seawater (FSW). The spawning mussel was transferred to a new 2 l glass beaker-containing FSW. The available generative cells were collected from the glass beakers for fertilization. Sperm and eggs were incubated for 20 minutes to fertilize them. A nylon plankton net (mesh size: 20 μm) was used to collect and wash fertilized eggs to remove excess of sperm. Fertilized eggs were incubated at 18 °C steadily at dark. After two days of incubation without feeding, a swimming straight-hinge veliger larval stage was obtained. Veliger larvae were cultured at glass beakers at density of 5 larvae ml^−1^. Each larva was fed with a diet of *Isochrysis zhanjiangensis* at 1 × 10^4^ cells every day. Fresh FSW was used to change culture water every two days, until the larvae developed into a pediveliger stage. This stage was used for the settlement and metamorphosis bioassays (see below).

### Settlement and metamorphosis bioassays

The settlement and metamorphosis bioassay was performed in glass Petri dishes (Ø 64 × 19 mm) (ASONE Corporation, Japan) according to the previous publications^19^. Each dish contained twenty competent pediveliger larvae, 20 ml AFSW and a developed BF (see above). Petri dishes were incubated at 18 °C without light for 48 h, and larvae were observed using a stereoscopic microscope (Olympus, Japan) at 1000 × magnification. The number of settled and metamorphosed larvae was counted and later expressed as percentages. “Settled larvae” stop swimming in water, settle and crawl on the BFs. “Post-larvae” changed both of larval behavior and the body restructure, including the settlement of larvae, the losing of velum, the development of gills, and the produce of juvenile/adult shell-dissoconch. Alive larvae that were lying on the bottom and not moving were defined as “Lying”. In opposite if larvae were crawling on the bottom they were group into the “Crawling” category. If larvae were not responding to the gentle needle touch and only had transparent shells, they were defined as “Dead”. A sterilized, clean, BF-free glass slides were placed in Petri dishes and used as negative controls. Each treatment contained nine replicates.

### The collection of surface-bound products

The bacterial solution (1 × 10^7^ cells ml^−1^) of *P. marina* was added into sterilized glass Petri dishes. BFs were formed at the bottom of glass Petri dishes at 18 °C for 48 h without light. Surface-bound products of *P. marina* BFs were collected followed a modified approach of Bao *et al*.^37^. BFs were scraped from 800 dishes (the total area is 2.3 × 10^4^ cm^2^) and suspended in 1 × PBS (Phosphate Buffer Saline, pH = 7.4). After centrifugation (12,000 g, 20 min), the precipitate was discarded, and the supernatant was filtered using 0.22 μm-pore size filter (Sangon Biotech Co., Ltd, Shanghai, China) to remove bacterial cells. The filtrate containing surface-bound products was transferred to 3.5 KDa cut off dialysis bags (MYM Biological Technology Company Limited, USA) to remove salts. The dialysis bag was incubated for 24 h at the room temperature. Surface-bound products were collected from the bag and were freeze-dried.

Different concentrations (0.5, 5, and 50 mg l^−1^) of surface-bound products were prepared by mixing with AFSW. Each Petri dish (Ø 64 × 19 mm) (ASONE Corporation, Japan) contained 20 milliliters of surface-bound products solution and twenty pediveliger larvae. Larvae were observed using a stereoscopic microscope (Olympus, Japan) at 1000 × magnification as mentioned above in the settlement and metamorphosis bioassay. Petri dishes containing AFSW (without BF and extracellular products) were served as a negative control. *P. marina* BFs were served as positive controls. Each tested concentration and control group set had six replicates. The percentages of post-larvae and larvae in different behavior group were recorded after 24 h, 48 h, 72 h, and 96 h.

### Scanning electron microscope (SEM)

*P. marina* BFs formed at the initial concentration of 1 × 10^7^ cells ml^−1^ for 48 h. BF was washed three times with AFSW and fixed overnight by 5% formaldehyde solution. Fixed BFs were washed by 1 × PBS solution three times to remove residual formalin. BFs were dehydrated by alcohol series and then dried at a room temperature. Finally, BFs were gold coated and visualized with a Hitachi S-3400 field emission SEM at 4 KV of accelerating voltage and magnification of 6500 × and 2700 × .

### Biofilms stained with fluorescent dyes

BFs were stained following the method of González-Machado *et al*.^49^. Extracellular products of BFs were stained by fluorescent dyes and observed using confocal laser scanning microscopy (CLSM). The following dyes were used: the concanavalin A, tetramethylrhodamine conjugate (ConA-TMR) (Invitrogen Company, Germany) for α-polysaccharides at Ex/Em = 552 nm/578 nm, calcofluor white M2R (CFW) (Sigma Company, United States) for β-polysaccharides at Ex/Em = 254 nm/432 nm, fluorescein isothiocyanate isomer I (FITC) (Sigma Company, United States) for proteins Ex/Em = 495 nm/519 nm, and DiIC18(5) oil, 1,1′-dioctadecyl-3,3,3′,3′-tetramethylindodicarbocyanine perchlorate (DiD’oil) (Invitrogen Company, Germany) for lipids (Ex/Em = 648 nm/670 nm). The concentrations of working solution were: ConA-TMR (10 mg dissolved in 2 ml of 8.4 mg ml ^−1^ NaHCO_3_ solution) at 944.8 μg ml^−1^, CFW at 189 μl ml^−1^, FITC (2 mg dissolved in 100 μl absolute ethanol) at 46.6 μg ml^−1^, DiD’oil (25 mg dissolved in 2.5 ml of absolute ethanol) at 79.4 μg ml^−1^. Working solutions were prepared by combining a dye with 150 mM NaCl. The *P. marina* BFs were stained in the dark for 20 min and then BFs were washed with NaCl at 150 mM to remove extra dyes. Stained BFs were observed by CLSM (see below). Three biological repetitions were set for each dye.

### Analysis CLSM images

The Leica TCS SP8 CLSM and the LAS X Version (Leica, Germany) software were used for acquisition of CLSM images. The 63 × (1.4 NA) objective was used for channel mode visualization. Nine sets of images (1024 × 1024 pixels, pixel size = 180.38 nm × 180.38 nm, Image size = 184.52 μm × 184.52 μm, z-step = 0.20 μm) were acquired from three random visual fields in three independent tested *P. marina* BFs. The threshold value was used to calculate the biovolume of the BFs using the Image J software (National Institutes of Health, United States). Biovolumes represented the amount of the individual components of extracellular products of BFs (μm^3^).

### Treatment of *P. marina* biofilms

*P. marina* BFs were treated with different agents according to previous studies^14^. Formalin-treated BFs were prepared by immersing BF in 5% formalin solution for 24 h. The UV-treatment BF were prepared by exposure under 5 W m^−2^, 254 nm of UV radiation for 1 h. The ethanol-treatment was prepared by immersing BFs in 10% or 100% ethanol solution for 0.5 h. The heat-treatment was prepared by heating BFs at 40 °C or 100 °C for 0.5 h. The antibiotic-treatment was prepared by immersing BFs in different concentrations of antibiotic solutions (0.1 A, 1 A, and 10 A) for 2 h. The 0.1 A antibiotic solution was a mixture of the penicillin G potassium (0.1 mg ml^−1^) and streptomycin sulfate (0.1 mg ml^−1^), 1 A and 10 A were ten-fold and hundred-fold concentrated solutions of 0.1 A. In order to remove remaining chemicals (formalin, ethanol, and antibiotics), treated BFs were washed nine times using 20 ml of AFSW. After the treatment of UV and heat, BFs were rinsed with 20 ml of AFSW for three times.

To investigate the bioactive moiety of the inductive cues of *P. marina* BFs, different enzymes treatment were performed following previously published method^65,66^. The enzymes’ stock solutions were prepared using mixture of the Tris-HCl (10 mM, pH = 7) and 50% glycerol. Working solutions were diluted with AFSW. The following concentrations of enzymes were used: trypsin (from bovine pancreas) − 0.01 mg ml^–1^, papain (from papaya latex) − 0.2 mg ml^–1^, β-glucuronidase (from bovine liver) − 0.01 mg ml^–1^, sulfatase (from *Patella vulgata*) − 0.5 mg ml^–1^, and α-amylase (from *Bacillus* sp.) − 0.01 mg ml^–1^. The lipase (from *Thermomyces lanuginosus*) was diluted using Tris-HCl (10 mM, pH = 7) to working concentration of 5 μg ml^–1^. All enzymes were obtained from Sigma (USA). The enzyme-treatment was incubating BF at enzyme solution at 25 °C for 3 h. BFs were washed with AFSW three times to remove extra enzyme and then were used to larval settlement and metamorphosis bioassays (see above).

### Rates of survive and dead cell of treated biofilms

Rates of living and dead cells of BFs were examined following previously published methods^14^. BFs were stained by the 5-cyano-2, 3-ditolyl tetrazolium chloride (4 mM) and the 4′, 6-diamidino-2-phenylindole (1 μg l^−1^). Dead cells and living cells were observed by Olympus BX51 epifluorescence microscope under excitation/emission = 365/455 nm and 1000 × magnification. Ten random views of three different BFs were used to calculate survival rate of cells of BFs.

### Data analysis

The percentage of post-larvae in settlement and metamorphosis bioassay was arcsine transformed to improve normality of data. Before statistical analysis of all data, normality of data was tested by Shapiro-Wilk’s W test. The Kruskal-Wallis followed by the Steel-Dwass All Pairs test was used to determine significant differences. The correlations analysis was performed by Spearman’s rank correlation test. Data processing was performed by JMP^TM^ software (version 10.0.0) and *p* < 0.05 was counted as cut-off for significant differences.

## Supplementary information

Supplementary information.
