# Supplementary material for: Monospecific Biofilms of Pseudoalteromonas Promote Larval Settlement and Metamorphosis of Mytilus coruscus
Source: Sci Rep. 2020 Feb 13;10:2577. doi: 10.1038/s41598-020-59506-1 (PMC7018757; doi:10.1038/s41598-020-59506-1)
Supplement: Supplementary file 1 — Supplementary information. [file 41598_2020_59506_MOESM1_ESM.doc]

**Monospecific Biofilms of *Pseudoalteromonas* Promote Larval Settlement and Metamorphosis of *Mytilus coruscus***

Li-Hua Peng ^1, 2, 3$^, Xiao Liang^1, 2, 3$^, Jia-Kang Xu ^1, 2, 3^, Sergey Dobretsov ^4,5*^, Jin-Long Yang^1, 2, 3*^

*^1^ International Research Center for Marine Biosciences, Ministry of Science and Technology, Shanghai Ocean University, Shanghai, China*

*^2^ Key Laboratory of Exploration and Utilization of Aquatic Genetic Resources, Ministry of Education, Shanghai Ocean University, Shanghai, China*

*^3^ National Demonstration Center for Experimental Fisheries Science Education, Shanghai Ocean University, Shanghai, China*

*^4^ Department of Marine Science and Fisheries, College of Agricultural and Marine Sciences, Sultan Qaboos University, Muscat, Oman*

*^5^ Center of Excellence in Marine Biotechnology, Sultan Qaboos University, Muscat, Oman*

* Corresponding authors. E-mail: jlyang@shou.edu.cn; sergey@squ.edu.om

Tel: + 86-21-61900403; Fax: + 86-21-61900405

^$^ These authors contributed equally.


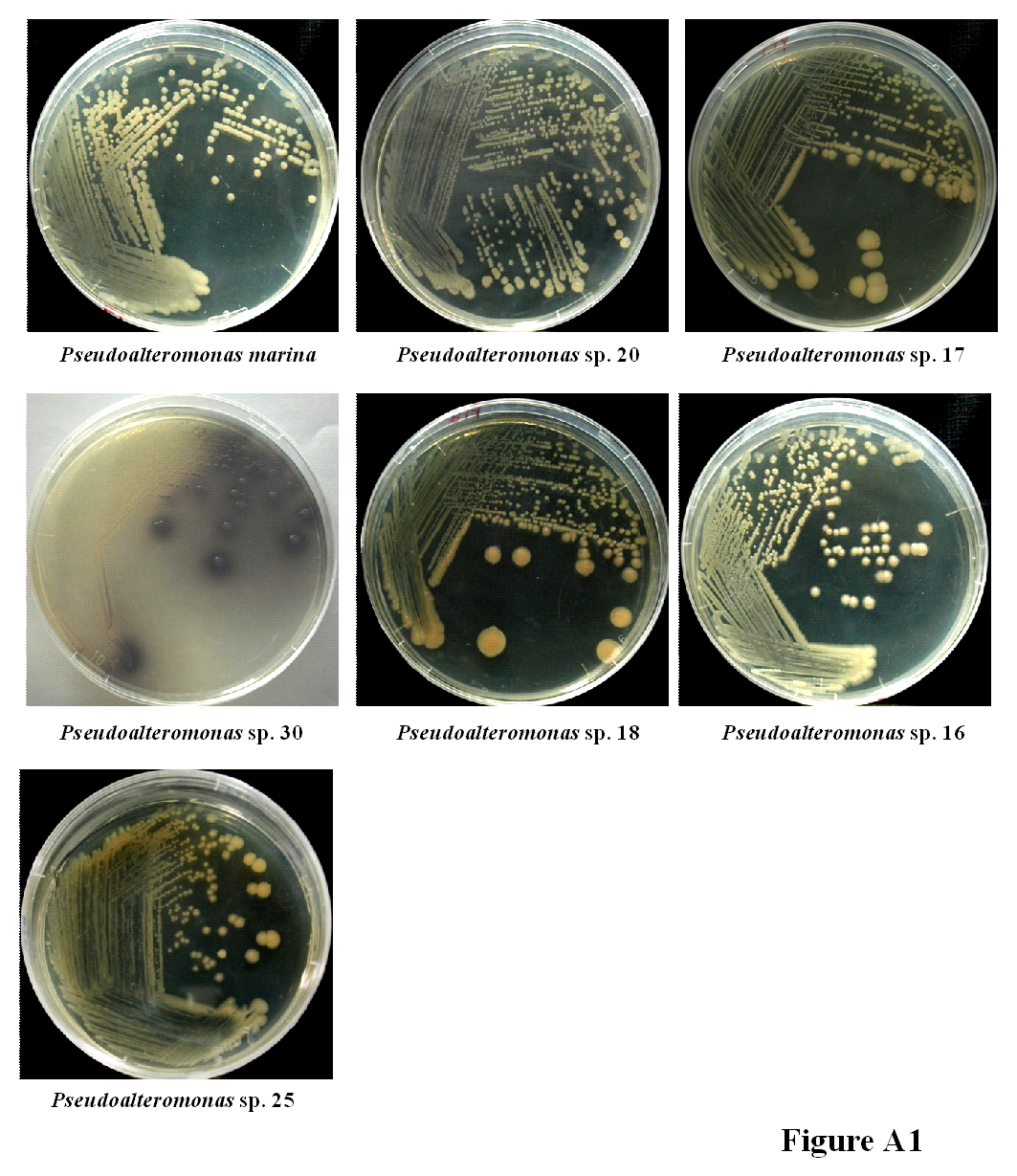


**Figure S1. The phenotype of *Pseudoalteromonas* strains.**

**
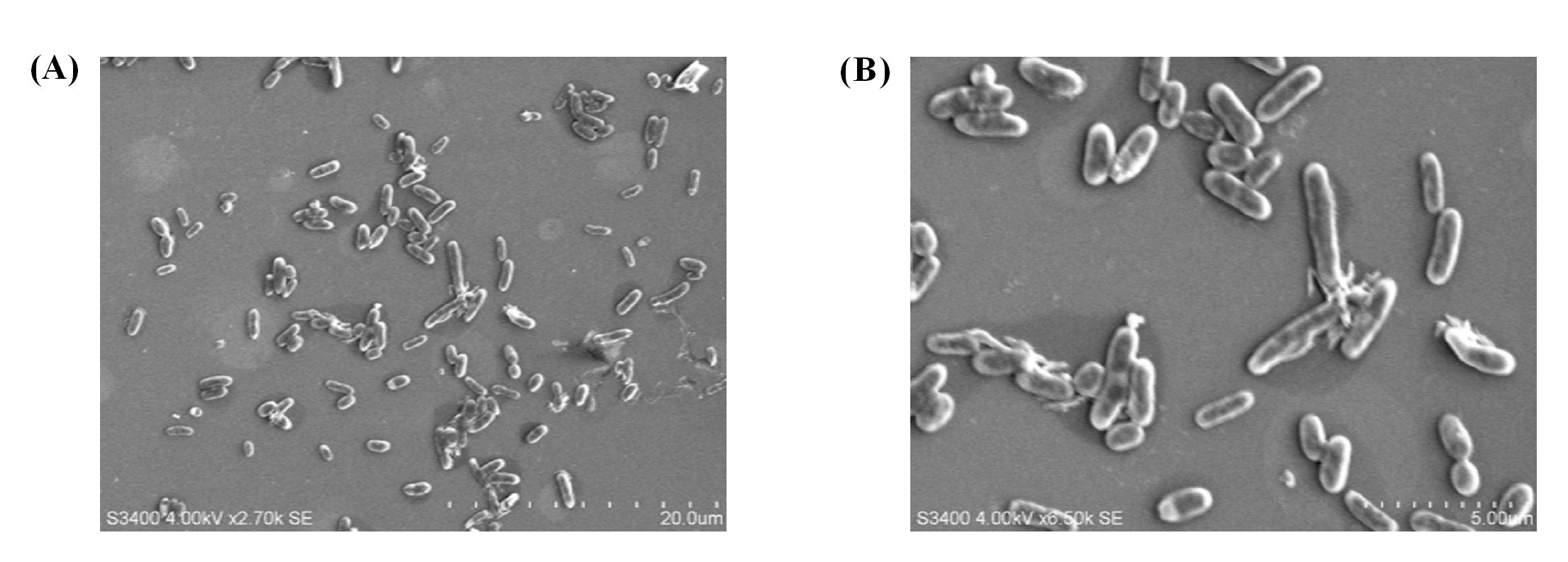
**

**Figure S2. The SEM image of *P. marina* BF with the scale bar of (A) 20 μm and (B) 5 μm.**

| Isolate | Accession no. | BLAST closest match | Accession no. of closest match | Similarity % | Isolated source | Source |
| --- | --- | --- | --- | --- | --- | --- |
| ECSMB14103 | JX206469 | *Pseudoalteromonas marina* | AY563031 | 99 | Glass slide | Peng et al. 2018 |
| ECSMB83 | MG967657 | *Pseudoalteromonas* sp. 15 | FR695483 | 99 | Glass slide | This study |
| ECSMB85 | MG967662 | *Pseudoalteromonas* sp. 17 | CP023398 | 99 | Glass slide | This study |
| ECSMC30 | KX099925 | *Pseudoalteromonas* sp. 30 | NR113971 | 99 | Gut | This study |
| ECSMB86 | MG967664 | *Pseudoalteromonas* sp. 18 | MF359415 | 99 | Glass slide | This study |
| ECSMB84 | MG967661 | *Pseudoalteromonas* sp. 16 | MG799503 | 99 | Glass slide | This study |
| ECSMB87 | MG967663 | *Pseudoalteromonas* sp. 25 | KY744378 | 99 | Glass slide | This study |

**Table S1. 16S rRNA gene sequence analysis of *Pseudoalteromonas* strains.**
